# Adverse Effects of Non-Metallic Nanoparticles in the Central Nervous System

**DOI:** 10.3390/ma16237264

**Published:** 2023-11-21

**Authors:** Katarzyna Sikorska, Krzysztof Sawicki, Magdalena Czajka, Lucyna Kapka-Skrzypczak, Marcin Kruszewski, Kamil Brzóska

**Affiliations:** 1 Centre for Radiobiology and Biological Dosimetry, Institute of Nuclear Chemistry and Technology, Dorodna 16, 03-195 Warsaw, Poland; m.kruszewski@ichtj.waw.pl (M.K.); k.brzoska@ichtj.waw.pl (K.B.); 2 Department of Molecular Biology and Translational Research, Institute of Rural Health, Jaczewskiego 2, 20-090 Lublin, Poland; 3 World Institute for Family Health, Calisia University, Nowy Swiat 4, 62-800 Kalisz, Poland

**Keywords:** nanoparticles, central nervous system, human health, neurodegenerative disease

## Abstract

The interest in nanoparticles (NPs) and their effects on living organisms has been continuously growing in the last decades. A special interest is focused on the effects of NPs on the central nervous system (CNS), which seems to be the most vulnerable to their adverse effects. Non-metallic NPs seem to be less toxic than metallic ones; thus, the application of non-metallic NPs in medicine and industry is growing very fast. Hence, a closer look at the impact of non-metallic NPs on neural tissue is necessary, especially in the context of the increasing prevalence of neurodegenerative diseases. In this review, we summarize the current knowledge of the in vitro and in vivo neurotoxicity of non-metallic NPs, as well as the mechanisms associated with negative or positive effects of non-metallic NPs on the CNS.

## 1. Introduction

The unique features of nanoparticles (NPs), e.g., size and strong absorption properties, make them more reactive in the biological environments, due to their exceptional chemical properties and ability to get different sites of organisms, as compared to a bulk material with the same chemical composition. To exploit these new capabilities, NPs were introduced to everyday life as industry and medicine products, e.g., biosensors, biomaterials, tissue engineering systems, drugs and drug-delivery systems for diagnosis and treatment of many diseases, including that of the central nervous system (CNS) [1,2,3,4,5,6,7].

The CNS can be reached by NPs through three different pathways. The first and the most likely is through systemic blood circulation, as NPs can cross blood–brain and blood–spinal cord barriers. The second path is a nose-to-brain route along nerve bundles that cross the cribriform plate to the olfactory bulb. Finally, NPs can diffuse through the nasal cavity mucosa to reach the branches of the trigeminal nerve in the olfactory and respiratory regions and then reach the brain stem via axonal transport [8,9,10].

**Figure 1:**
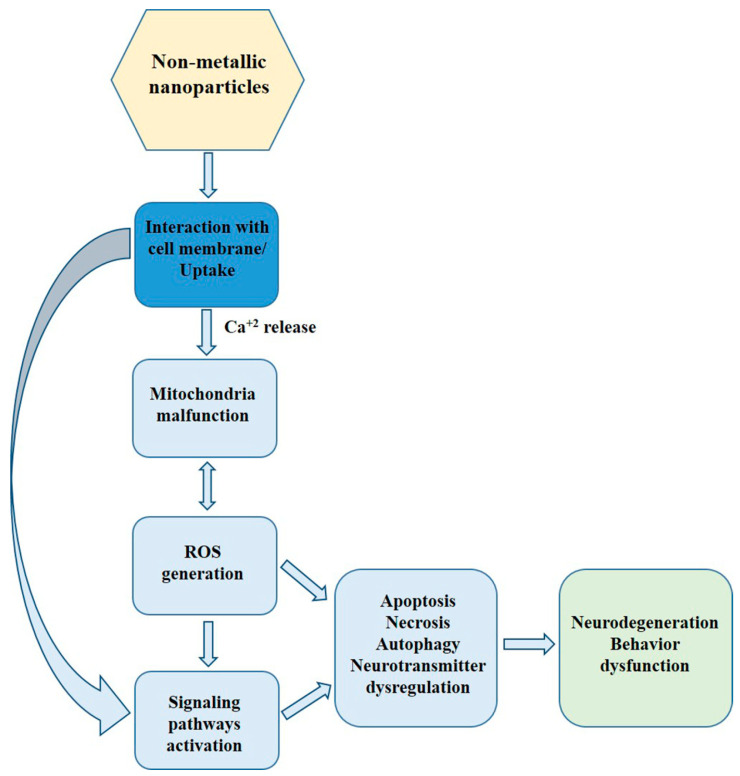
The scheme of direct and indirect action of nmNPs in CNS.

The presence of nanomaterials in the CNS may have unexpected consequences, including acute or chronic neurological complications [2,8]. Though numerous studies clearly indicate in vitro and in vivo oxidative stress-associated adverse effects of metallic NPs in the CNS (for recent review, see Sawicki et al. [11]), the toxicity of non-metallic NPs (nmNPs) in the CNS remains obscure. Here, we review the in vitro and in vivo studies regarding the neurotoxicity of nmNPs and summarize the current state of knowledge in this area (Figure 1).

## 2. Non-Metallic NPs

Pure carbon NPs constitute the largest and most important group of nmNPs, which include nanotubes, nanohorns, nano-onions, nanodiamonds, fullerenes and graphene, and exhibit large diversity in structure, morphology, physical properties and chemical reactivity [1,12,13,14,15]. Another carbon-containing, large group of nmNPs are dendrimers. These NPs have a highly branched three-dimensional structure, consisting of an initial core, several internal layers, repetitive units and several terminal active surface groups. The presence of a hydrophobic core and multiple surface groups makes them a good candidate for high-load drug carriers [1,16,17]. Other nmNPs discussed later in this review include polymer NPs, silica NPs, apatite, and hydroxyapatite NPs, also widely described in scientific literature in connection with CNS.

**Figure 2:**
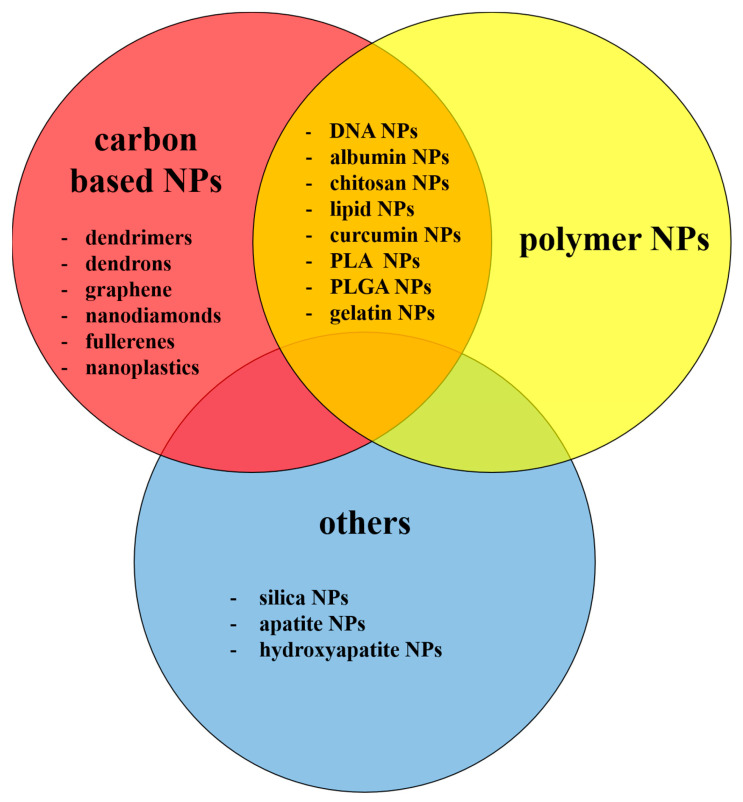
Types of the nmNPs discussed in the present review.

These and other types of NPs discussed in the present review are summarized in Figure 2.

## 3. Uptake of Non-Metallic NPs

### 3.1. Uptake of Non-Metallic NPs by Normal CNS-Derived Cells In Vitro

Various cells responsible for maintaining brain homeostasis can accumulate nmNPs. The most important seems to be microglia, brain macrophages responsible for clearance from damaged or dead cells and other dangerous particles [18]. Microglia are also primary cells responsible for the clearance of multi-walled carbon nanotubes (MWNTs), an allotropic form of pure carbon structured in a shape of empty nested fibers (concentric cylinders). MWTNTs distinguish from other types of nanomaterials, as their length can be thousands of times larger than their diameter [19]. Carboxylic acid group modified MWNTs were observed inside microglial cells in ex vivo mixed cultures isolated from the rat’s brain [20]. In vitro, an average phagocytosis time for MWNTs by the human microglia (BV-2 cells) was approximately 2 h, and MWNTs were completely internalized after 6 h [21]. In line, the presence of acid-oxidized or pristine MWNTs in N9 microglial cells was observed after several hours of treatment [22]. The MWNTs accumulated in various cellular compartments, such as cytoplasm, phagolysosomes, endosomes and lysosomes, with the exception of the nucleus, which seemed to be free from MWNTs [20,22]. With regard to other types of nmNPs internalized by microglial cells, the following can be listed: silica nanoparticles (SiNPs) [23], indocyanine green (ICG)-coated polycaprolactone (PCL)-rhodaminedoped NPs, Si-ICG/PCL-polylactic acid (PLLA)-rhodamine-doped NPs [24] and G4 and G4-C12-modified polyamidoamine (PAMAM) dendrimers [25]. The internalization of cyanine5-labeled hydroxyl terminated 4th generation (G4) PAMAM dendrimers by microglial cells was more efficient than by other types of brain cells, such as astrocytes, oligodendrocytes or neurons [26,27,28,29,30].

Besides microglia, the ability to internalize nmNPs has been shown for astrocytes, which are involved in many processes, including the biochemical support of endothelial cells, the formation of blood–brain barrier (BBB), the provision of nutrients to the nervous tissue, maintenance of extracellular ion balance and repair and scarring process of the brain [31]. The astrocytes internalized MWNTs, less efficiently than microglial cells [19]; however, the process was facilitated by the functionalization of MWNTs with amine groups [32].

The internalization of nmNPs by neurons, paramount cells in the CNS, is less efficient when compared to the other cell types. Resveratrol-loaded NPs based on poly(N-vinylpyrrolidone)-b-poly(ε-caprolactone) (PVP-b-PCL), were mainly localized in the cytoplasm, dendrites and axons of cortical neurons [33]. In line, neurons internalized also polylactide-co-glycolic-acid (PLGA) NPs loaded with curcumin and modified with g7 ligand [34], poly(lactide-co-glycolide)-block-poly(ethylene glycol) (PLGA-PEG) conjugated with B6 peptide and loaded with curcumin [35] or lactose myristoyl carboxymethyl chitosan and algal polysaccharide myristoyl carboxymethyl chitosan NPs [36]. In addition, mouse hippocampal neurons and mouse dorsal root ganglion neurons internalized nanodiamonds and accumulated them inside the cell bodies of cortical neurons and inside membrane-surrounded organelles [37]. It was also shown that PAMAM dendrimers were able to penetrate into human neural progenitor cells cultured as a 3D neurosphere model [38] and that amino-functionized G5 PAMAM dendrimers accumulate in the plasma membrane of neurons, as soon as 30 min after treatment [39].

Finally, the presence of nmNPs was also reported for brain endothelial cells. This type of cell maintains the delicate balance of ions, nutrients and other molecules essential for proper brain function and is responsible for removing toxins from the CNS [40]. Liu et al. [41] showed that SiNPs modified with PEG were taken up by mouse cerebral endothelial cells (bEnd.3) with the efficiency dependent on the size of NPs. This was confirmed by Ye et al. [42], who reported that the uptake of SiNPs by human capillary microvascular endothelial cells (hCMEC/D3) was more efficient for 50 nm NPs than for 200 nm NPs. SiNPs were localized inside intracellular vesicles along the endo–lysosomal pathway, inside membrane-bound vesicles and in late endosomes. Other kinds of nmNPs taken up by brain endothelial cells include PEG and polyethylenimine nanogel [43], amino-functionalized MWNTs [32] and fullerenes [44]. The interaction of MWNTs with the plasma membrane of endothelial cells was observed after 4 h of incubation, while their accumulation in endoplasmic vesicles and multi-vesicular bodies was observed after 24 h of treatment and was depended on the NPs concentration [32].

### 3.2. Uptake of Non-Metallic NPs by CNS-Derived Cancer Cells In Vitro

Though NP uptake by normal brain cells may have adverse effects, the internalization of NPs by brain cancer cells opens the possibility for the development of new NP-based theranostic therapies. Among several types of brain cancers, neuroblastoma and astrocytoma are the most frequent ones [45,46] and are often used for testing their interactions with NPs. Various neuroblastoma and astrocytoma cells were able to take up nmNPs, including SiNPs [47], rhodamine doped Si-ICG/PCL/PLLA NPs [24], curcumin-loaded lactoferrin NPs [48], curcumin-loaded PLGA NPs [49], carbon nanotubes (CNTs) [50], and polymeric NPs and liposomes [51]. These particles accumulated in a membrane region, cytoplasm and a region over the nucleus [47,49,50]. According to Listik et al. [51], the efficiency of internalization depends on the type of NPs and cancer cells. The authors studied an uptake of polysorbate-80 coated polymeric NPs and liposomes coupled with G-protein estrogen receptor selective agonist, in N2a and SHSY5Y neuroblastoma cells. Liposomes were identified inside N2a cells after 6 and 24 h of incubation, while polymeric NPs were detected only after 24 h. In the case of SHSY5Y cells, liposomes were internalized in less than 30 min, whereas the internalization of polymeric NPs is characterized by a slower kinetic profile (internalization was observed after more than 6 h).

Gliomas, which arise from glial cells of the brain or spine and are one of the most invincible cancers, constitute another important group of brain cancers [52]. Many studies indicated that nmNPs were easily taken up by glioma cells, e.g., C6 cells. The NPs tested in this in vitro model include G7 PAMAM dendrimers with amine, acetamide and carboxylate end groups [53], coumarin-6 loaded D-α-tocopheryl PEG1000 succinate (TPGS) coated liposomes [54], PEG-PCL NPs loaded with resveratrol [55], curcumin-loaded polysaccharide nanoformulations based on hyaluronic acid and chitosan hydrochloride NPs [56], temozolomide-loaded PLGA NPs functionalized with anti-EPHA3 [57], and transferrin-conjugated polylactide (PLA)-D-α-tocopheryl-PEG-succinate diblock copolymer NPs [58]. The uptake of NPs occurs very fast; the NPs were observed inside glioma C6 cells after 2 h of incubation [59]. Moreover, it was shown that modifications of NPs and/or targeting with receptor-specific antibody (ephrin type-A receptor 3 tyrosine kinase antibody-modified PLGA NPs and enhancer-modified albumin NPs) change internalization efficacy [57,60].

### 3.3. Uptake of Non-Metallic NPs by CNS In Vivo

In order to reach the brain, NPs must cross the BBB, a decisive barrier between the brain and systemic circulation. This structure is formed by a complex system of endothelial cells, astroglia, pericytes and perivascular mast cells and is stabilized by a multiprotein complex that seals gaps between endothelial cells and prevents leakage of the barrier, known as a tight junction. The BBB plays an important metabolic role by disposing of waste products, metabolizing different chemical compounds, both drugs and toxins, and protecting the CNS from changes in the ionic composition of cerebrospinal fluid [61,62].

NPs can penetrate through the tight junctions of BBB and their presence in brain cells and tissue was described for single-walled carbon nanotubes (SWNTs) [63], cationic albumin conjugated PEG–PLLA NPs [64], SiNPs [65] and many others. Translocation from systemic circulation to brain tissue occurred very fast. Labeled, amino-functionalized MWNTs were present in the brain of mice 5 min after intravenous injection [32]. Similarly, poly(n-butylcyanoacrylate) dextran polymer NPs coated with polysorbate 80 were detected in mice brains 18 min after injection [66]. The mechanisms of NP uptake by brain cells include phagocytosis, macropinocytosis, and clathrin and caveolin-mediated endocytosis [25,67]. Different receptors may also be involved, as was shown for folic acid [68], apolipoprotein E, low-density lipoprotein receptors and GLUT transporter [67,69].

Initial localization of NPs in the brain tissue depends mainly on a mode of administration. Intravenously administered amino-functionalized MWNTs localized mainly in brain capillaries and parenchyma fraction [32], whereas radiolabeled SiNPs modified with aminopropyltriethoxysilane applied to mice through intranasal instillation localized mostly in the striatum. From brain microvasculature, NPs can be transferred to other regions of the brain, such as the hippocampus (CA1 and CA3 regions), brain stem, cerebellum, and frontal cortex, while intranasal administration resulted in the presence of NPs also in the olfactory bulb [65]. The final localization of NPs depends mostly on their modifications and mode of administration. Bardi et al. [70] tested the internalization of oxidized and non-oxidized amino-functionalized MWNTs (oxMWNT-NH_3_^+^ and MWNT-NH_3_^+^, respectively) and reported that, after stereotactic administration, MWNTs-NH_3_^+^ were abundantly and evenly dispersed along the injection site and within the brain parenchyma, while oxMWNTs-NH_3_^+^ were observed mainly as small clusters in intracellular vesicles in microglia, astrocytes and neurons, with the minority in extravesicular cytoplasmic or brain parenchymal areas. After intracortical administration, MWNTs-NH_3_^+^ were dispersed throughout the brain parenchyma, forming small aggregates inside membranous intracellular vesicles or as a single nanotube residing in the cytoplasm. In contrast, oxMWNTs-NH_3_^+^ were present in clusters and were rarely seen in the cytoplasm, as a single nanotube.

Despite NP modifications and mode of administration, NP uptake and localization in the brain tissue depend also on their size, coating and cargo. Size-dependent differences in the uptake and biodistribution of nmNPs were reported for fluorescent polystyrene latex nanospheres. 20 nm fluorescent polystyrene latex nanospheres were not detected in the brain of rats after intravenous injection or oral pharyngeal aspiration, whereas 100 nm and 1000 nm spheres were present in the CNS 24 h after administration [71]. Similarly, the study with hydroxyl-PAMAM dendrimers in a dog model of a hypothermic circulatory arrest revealed that G6 dendrimers (approx. diameter 6.7 nm) showed extended blood circulation time and increased accumulation in cerebrospinal fluid (CSF), hippocampus, cerebellum and cortex of the injured brain, whereas smaller, G4 dendrimers (approx. diameter 4.3 nm) were undetectable in the brain even 48 h after the final administration [30].

It was also shown that coating with a ligand for the low-density lipoprotein receptor-related protein-1 peptide (angiopep-2), antibodies, bovine serum albumin, apolipoprotein E, and transferrin improve the NP uptake by CNS. MWNTs coated with angiopep-2 were more intensively taken up by glioma-bearing brains than NPs without the ligand [72]. Enhanced accumulation in brain tissue was also observed for transferrin-loaded solid lipid NPs [73], liposomes with bovine serum albumin [74], biodegradable polymersomes with cationic bovine serum albumin [75] and antibody-coated polymer-based NPs [76]. This effect was attributed to enhanced phagocytosis by macrophages [76]. Enhanced uptake of NPs into the brain was also observed after the coating of NPs with a nonionic surfactant—polysorbate 80, nonionic block copolymer—poloxamer 188 and chitosan polysaccharide [77]. In line, Calvo et al. [78] reported that PEGylated poly(cyanoacrylate) NPs can penetrate the brain of mice and rats. The concentration of PEGylated NPs was significantly higher than uncoated ones in the majority of brain structures, especially those in the deeper regions of the brain (striatum, hippocampus, hypothalamus and thalamus). Further, fluorescent COOH-modified polystyrene particles covalently modified with methoxyPEG(NH_2_) rapidly diffused within normal rat brain tissue, but only if coated with an exceptionally dense layer of PEG [79].

An Important factor affecting the penetration of NPs is also the brain intactness. The ability of G6 dendrimers to cross the BBB correlated with the extent of CNS inflammation, while their accumulation was more efficient in injured places [30]. Similarly, in a rabbit cerebral palsy model, ethylenediamine-core PAMAM dendrimers were more abundantly present in the brain parenchyma of regions characterized with significant inflammation, as compared to the healthy regions [26].

## 4. Toxicity of Non-Metallic NPs in Mammals

### 4.1. In Vitro and Ex Vivo Toxicity

Many reports indicate that metallic NPs are toxic to CNS. Their neurotoxicity is associated with the induction of oxidative stress in the brain tissue through the release of metal ions [11]. Similarly, numerous reports also show detrimental effects of nmNPs (Table 1).

The tables included in this publication present information on the toxic and non-toxic effects of various types of NPs on the CNS (in vitro and in vivo studies).

In vitro research on various types of brain cells clearly show that toxicity of nmNPs is dependent on cell type and uptake of NPs. In accordance, Du et al. [81] observed an intense uptake of SiNPs by microglial N9 and endothelial bEnd.3 cells, while neuronal HT22 cells barely internalized the NPs. Consequently, toxicity of SiNPs described as morphological changes, such as swelling and cell membrane blebbing, was higher in BV-2 and N9 cell lines than in HT22 cells. A similar result was reported for CNTs, as sensitivity of internalization prone microglia to CNTs was higher than internalization tardy neurons [19].On the other hand, many publications reported also the lack of detrimental effects of nmNPs. In the study conducted by Ducray et al. [24] exposure of primary hippocampal cultures to SiNPs coated ICG/PCL-rhodamine-doped NPs or Si-ICG/PLLA-rhodamine-doped NPs did not affect cell viability, however as noticed earlier, these NPs impaired cell differentiation. The lack of impact of SiNPs on cellular viability was also observed for endothelial cells (bEnd.3) [96] and for A-172 brain cells [105]. In addition, carbon NPs [19,21,22,32,44,97], modified PAMAM dendrimers [28,89,106], PEG–based dendrimers [107], polymer NPs [102], PEG/polyethylenimine (“nanogel”) NPs modified with oligonucleotides [43], PLGA NPs modified with PEG and phage-displayed peptides [101], solid lipid NPs, as well as albumin and chitosan NPs [67,75,91,108] were also reported to be non-toxic to glial cells, neurons and endothelial cells.

Toxicity of nmNPs to CNS-derived normal cells is definitely an unfavorable phenomenon. On the other hand, toxicity to cancer cells may be exploited for development of new anticancer drugs provided that selective accumulation of NPs in cancer cells can be achieved. Numerous reports indicate the toxic effects of nmNPs on glioma and glioblastoma cells in vitro. Transferrin conjugated PLA-D-α-tocopheryl PEG succinate diblock copolymer NPs [58] inhibited growth of glioma C6 cells. In line, exposure to PAMAM dendrimers and dendrons resulted in reduced cell size, rounded shape and loss of neurites of cells belonging to several glioma lines [109].

In contrast to the above results, several publications showed the lack of toxicity of nmNPs to CNS-derived cancer cells, e.g., MWNTs and SWNTs shown no toxicity to glioma GL261 [21,110], also no adverse effects were observed after treatment of glioblastoma A-172 cell line with SiNPs [105].

More detailed information about nmNPs effects on CNS-derived non-cancer and cancer cells in vitro is presented in Table 1 and Table 2.

### 4.2. Toxicity In Vivo

Many in vivo studies conducted using mammals describe neurotoxic effects associated with the presence of nmNPs in the brain (Table 3).

Interestingly, exposure to nmNPs might exert different effects in various brain regions, e.g., polysorbate 80-modified chitosan NPs were deposited mainly in the frontal cortex and cerebellum of the rats’ brain after systemic injection. Although no signs of oxidative stress were observed, apoptosis and necrosis of neurons and mild inflammatory response were observed in the frontal cortex, whereas in the cerebellum a decrease in the expression of glial fibrillary acidic protein was the only detected sign of degenerative changes [143]. In line, though reductions in the number and dispersion of Nissl bodies were observed in neurons of mice exposed to FITC-tagged SiNPs, the effect was spatially limited to the frontal cortex and was not present in the other brain regions, such as the hippocampus [137]. The toxicity of nmNPs partially depends on the disruption of BBB integrity, as acute pulmonary exposure to MWNTs caused a neuroinflammatory response in rodents that was dependent on the disruption of BBB integrity [141].

On the other hand, many in vivo studies show also that nmNPs are low- or non-toxic to CNS. Bardi et al. [97] demonstrated that the injection of MWNTs coated with pluronic F127 surfactant into mice brain resulted only in a small injury area. No damage to the overall brain structure and tissue was observed. No pathological changes were also observed in mice brain after 28 days of exposure to carboxylated MWNTs [147]. No adverse effects associated with CNS in rodents, despite the penetration of BBB, were also detected for other kinds of carbon NPs, such as fullerenes [148], dextran-coated graphene oxide nanoplatelets [163], water soluble fluorescent carbon nano onions [151] and nanodiamonds [37]. In line, similar results were published for other types of nmNPs, such as SiNPs [105], dendrimers, dendriplexes [90] and poly(n-butyl cyanoacrylate)dextran polymers [66]. See Table 3 for details.

## 5. Mechanism of nmNPs Toxicity. Other Adverse Effects of Non-Metallic NPs in Mammals

In spite of direct interactions with cells or cellular components leading to non-metallic nanomaterials toxicity, several indirect mechanisms were proposed to have a role in this process.

Non-toxic doses of SiNPs caused Ca^2+^ flux into neuronal cells in a size and surface charge dependent manner, stimulating long-lasting but reversible calcium signaling. Voltage-dependent and transient receptor potential-vanilloid 4 channels were involved in this process. Interestingly, NP internalization was not necessary to induce the calcium flux [83]. The enhancement of Ca^2+^ concentration after interaction with plasma membrane was also observed in pyramidal neurons and astroglial cells of rat hippocampal slices treated with 5th generation dendrimers (PAMAM G5). The increase in Ca^2+^ concentration was followed by the mitochondria depolarization of astroglial cells [39].

Another indirect mechanism of toxicity of non-metallic NPs involves alterations in gene and protein expression. Inhibition of the expression of mitochondrial deacetylase SIRT3 that plays an important role in regulation of cellular metabolism was proposed as a cause of increased oxidative stress in mitochondria and reprogramming of cellular metabolism in LPS-stimulated mice microglia treated with single-walled carbon nanohorns. This resulted in G1 arrest and increased the apoptosis of treated cells [87].

The treatment of rat brain capillary endothelial cells with gold and polymer-coated Si-ICG/PCL and Si-ICG/PCL/PLLA NPs resulted in a time- and concentration-dependent decrease in the phosphorylation of MAPKs, which participate in cellular response to a diverse array of stimuli, such as mitogens, osmotic stress and pro-inflammatory cytokines, and regulate cell proliferation, gene expression, differentiation, mitosis and many others [82]. Yet, another alteration in gene expression was shown in a 3D model of human neural progenitor cells incubated with PAMAM-NH_2_ dendrimers. In this model, NPs inhibited cell proliferation and migration, which was accompanied by the down-regulation of several genes, including early growth response gene 1 (EGR1), insulin-like growth factor-binding protein 3 (IGFBP3), tissue factor pathway inhibitor (TFPI2) and adrenomedullin (ADM) [38].

Incubation of neuroblastoma N2a cells with MWNTs promoted nuclear translocation and acetylation of NFκB transcription factor in a dose-dependent manner, followed by up-regulation of nNOS and an increase in NO production [111]. The NFκB-dependent signaling pathway is involved in the regulation of immunity, inflammation, cell differentiation, proliferation and apoptosis [164].

Another important cellular signaling pathway affected by nmNPs is the p53-mediated pathway. The incubation of PC12 cells, often used as a model of dopaminergic neurons, with SiNPs, caused an upregulation of p21 and GADD45A proteins that resulted in G2/M arrest and induction of apoptosis [65]. The upregulation of p21 and GADD45A proteins, accumulation of cells in the G2/M phase of cell cycle and induction of apoptosis were also observed in primary cultures of rats’ cortical neurons and N9 microglia cells after incubation with SiNPs [81].

Another mechanism that has been proposed to be responsible for adverse effects of nmNPs is alteration in redox balance. Apoptosis triggered by ROS production was proposed as a mechanism of SWNTs and graphene toxicities. ROS generation in PC12 cells treated with graphene layers [113] and SWNTs [114] resulted in the upregulation of caspase 3 apoptosis and was dependent on the PEG coating of nanomaterials. MWNTs were also able to induce alterations in the microenvironment and microstructure of brain tissue associated with NOS and ROS production. After treatment with MWNTs, high expression of nNOS and increased ROS production were observed in rostral ventrolateral medulla and nucleus tractus solitaries—two regions of the brain, which play important roles in the regulation of sympathetic nerve activity [111]. This result was confirmed by Zheng et al. [88] who proved that inhalation of MWNTs significantly alters the balance between the sympathetic and parasympathetic nervous system in rats. An enhanced ROS production and lipid peroxidation were also observed in rat hippocampus after exposure to SWNTs functionalized with PEG [142] and SiNPs [138]. The latter was confirmed by an increase in the expression of superoxide dismutase (SOD) and catalase (CAT) activity, two enzymes responsible for the removal of ROS [138].

Incubation of microglial BV-2 and N9 cells with MWNTs and single-wall carbon nanohorns resulted in a dose-dependent cell division arrest and apoptosis [86,87]. Similarly, the increase in apoptosis level was reported for neuronal NeuroScreen-1 cells (NS-1) incubated with MWNTs [85] or primary neuronal cells treated with G4-C12 PAMAM dendrimers [25].

Yet, another indirect effect mechanism leading to nmNPs toxicity in CNS-derived cells involves the release of pro-inflammatory cytokines that may led to inflammation in the brain. N9 microglial cells treated with SiNPs produced pro-inflammatory interleukin 1β (IL-1β) and N-terminal fragment of gasdermin D, a marker protein for pyroptosis [81], whereas nanoplastic treatment induced pro-inflammatory response in astrocytes, including the up-regulation of tumor necrosis factor alpha and IL-1β [165]. MWNTs caused an increase in TNF-α and IL-1β expression, indicating the pro-inflammatory action of nmNPs inside the brain (in vivo studies). The release of pro-inflammatory cytokines was also reported for shortened-by-oxidation, amino-functionalized MWNT (oxMWNTs-NH_3_^+^). Furthermore, oxMWNTs-NH_3_^+^ induced the higher-than-long, non-oxidized analog expression of GFAP and CD11b, which points to the more intense glial cell activity and degenerative changes [70]. An increase in the expression of TNF-α and IL-1β was also observed in the striatum of rats treated with SiNPs [65].

nmNPs also affected the functionality of neural cells, such as the differentiation and formation of neurite and dendrites. Treatment of SH-SY5Y cells with ICG/PCL-rhodamine-doped NPs and Si-ICG/PLLA-rhodamine-doped NPs resulted in a significant down-regulation of the expression of differentiation marker MAP-2 [24]. Accordingly, a study by Hollinger et al. [27] showed that incubation of rabbits primary mixed glial cells with 2-PMPA dendrimers triggered up-regulation of transforming growth factor beta (TGFβ), which plays an important role in the regulation and differentiation of immune and stem cells. Treatment of primary cultured cortical neurons with increased concentrations of SiNPs resulted in a statistically significant decrease in the number of dendrites. Furthermore, subsequent analysis showed that soma of the neurons collapsed and dendrites disappeared [80]. In line, reduction in the ability to form neurites after NGF stimulation was observed in PC12 cells treated with SiNPs modified with aminopropyltriethoxysilane, likely due to disorder in cytoskeletal structure [65].

The impact of nmNPs on neurotransmitter secretion was described by Wu et al. [65], who showed that exposure of rats to SiNPs caused a decrease in dopamine level in the striatum and in hippocampus. In line tissue, decreased levels of epinephrine, norepinephrine and dopamine was reported in the blood of mice exposed to MWNTs [111]. The negative impact of SiNPs on the functionality of dopaminergic neurons shows that these nmNPs can be a risk factor of neurodegenerative diseases. Further evidence that SiNPs might be at risk of neurodegenerative diseases was delivered by You et al. [137], who reported that phosphorylation Tau protein was significantly increased in the frontal cortex of SiNP exposed mice. It was accompanied by increased phosphorylation of ERK in the frontal cortex and hippocampus, and c-Jun N-terminal kinases (JNK) in the frontal cortex. The impairment of Tau protein phosphorylation is associated with neurodegeneration and contributes to the development of Alzheimer disease. The above-mentioned changes were associated with microglia activation and upregulation of pro-inflammatory markers, suggesting that exposure to SiNPs can lead to neuroinflammation that underlies many neurodegenerative disorders. The same work showed the significant impact of SiNP exposure on synapses function and structure. Exocytosis and endocytosis are essential processes in synapse firing, allowing communication to occur between neurons. It was found that exocytosis was significantly impaired in the SiNP-exposed mice in the frontal cortex. However, neither endocytosis nor exocytosis was affected in the hippocampus. Furthermore, after ex vivo exposure of primary cortical neurons to SiNPs, a decrease in the expression of synapsin I and a parallel increase in the expression of synaptophysin were observed, both proteins playing a pivotal role in the proper functioning of synapses.

Finally, nmNPs also affect the behavior of exposed animals. Treatment of mice with SiNPs via intranasal instillation resulted in mood dysfunction and cognitive impairment. Short-term memory and spatial learning, estimated by using a Morris water maze test, were impaired. Furthermore, the mice social interaction activity was decreased after 2 months of NP exposure; however, any symptoms of depression were not detected [137]. On the contrary, Wu et al. [65] reported that intranasal administration of SiNPs for 1 or 7 days did not result in any changes in animals’ behavior or cause histological changes in the brain tissue; however, it should be noted that dose used in this study was relatively low (20 μg/day) and exposition time was shorter, as compared to the study mentioned before. Adverse effects of nmNPs on rodent behavior was confirmed by the study of Dal Bosco et al. [142], who showed that the treatment of rats with SWNTs-PEG caused a significant deficit in the retrieval of fear memory.

## 6. Adverse Effects of Non-Metallic NPs in Non-Mammalian Organisms

Many types of nmNPs are released into the environment, as a result of their use, and may impact animals living there. Therefore, testing the toxicity of nmNPs on the CNS of higher non-mammalian organisms seems to be crucial for estimating of the environmental risk associated with their use.

Exposure of adult Japanese rice fish (*Oryzias latipes*) to fluorescent polystyrene NPs for 7 days revealed the presence of particles in the brain, indicating that nanoplastics have the innate capacity to cross the BBB [166]. In line, amino-modified polystyrene nanobeads were more prevalent than similar microparticles in the brain of exposed Crucian carp (*Carassius carassius*). The presence of polystyrene nano- and microparticles in the brain coincided with alterations in behavioral patterns, decreased brain mass and morphological changes in the cerebral gyri [167]. In line, 15 days of exposure to polyethylene nano- and microplastic caused varying degrees of necrosis, fibrosis, changes in blood capillaries, tissue detachment, edema, degenerated connective tissues, and necrosis in large cerebellar neurons and ganglion cells in the tectum of juvenile common carp (*Cyprinus carpio*). The changes were accompanied by a decrease in the activity of acetylcholinesterase (aChE) [168]. The gut–brain axis related toxicity of nanoplastic was recently reviewed, and it is clear that these particles may induce oxidative stress, disturb neurodevelopment, and impact behaviour and immune system activation [165].

The studies performed on bivalves and crustaceans confirmed the neurotoxic effect of nanoplastic. It was shown that nanoplastic can inhibit cholinesterase in the hemolymph of Mediterranean mussel (*Mytilus galloprovincialis*), an enzyme responsible for the breakdown of neurotransmitters [169]. The same effect was observed in brine shrimp (*Artemia fransiscana*) [170].

Despite nanoplastic, significant lipid peroxidation and GSH depletion were found in the brains of largemouth bass (*Micropterus salmoides*) after 48 h of exposure to uncoated fullerenes [171]. In line, 21 days exposure of zebrafish (*Danio rerio*) to fullerenes, short and long MWNTs and SWNTs, caused significant disturbances in lipid, revealed as an elevation of the lipid to protein ratio and in the brain and gills. In addition, a decrease in the level of unsaturated lipids was reported in the brain of fullerene exposed males. In contrast to the result obtained for male zebrafish, the level of unsaturated lipids in the brain in female fish exposed to fullerenes increased [172].

The zebrafish model was also used to study embryonic developmental toxicity of SiNPs. The results revealed persistent changes in larval behavior [173]. In agreement, transcriptomic analysis suggests neurodegeneration and motor dysfunction in larval zebrafish after polystyrene NP treatment. The authors clearly indicate the changes in behavior and physiology, potentially decreasing organismal fitness in contaminated ecosystems [174].

Studies on in vivo toxicity of different types of nmNPs in mammals and non-mammalian organisms are summarized in detail in Table 3 and Table 4.

## 7. Conclusions

In the past decade, rapid growth of interest in nanotechnology and increasing use of NPs in commercial applications have been widely observed. In general, despite being similar in shape and size, nmNPs seem to be less toxic than metal nanomaterials. The exact cause of the lesser toxicity of nmNPs over similar metallic ones is not yet explained. It might be speculated that the smaller density of nmNPs, as compared with the metallic ones, affects their interactions with cellular components, such as cell membranes, they might differ in composition and/or behavior to protein corona, or a smaller amount of metal ions is released from the NPs that might contribute to the generation of oxidative stress. Whatever it is needs further investigation; however, the impact of nmNPs on human health should not be disregarded.

**Figure 3:**
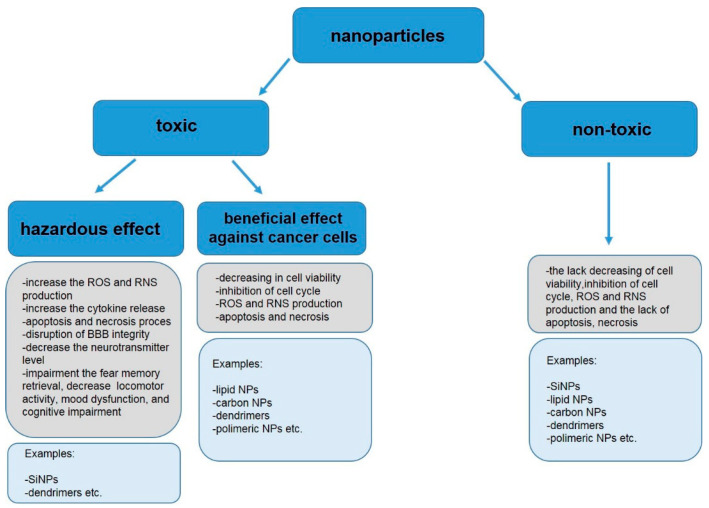
Effects of nmNPs in CNS.

The summary of current knowledge about the toxicity of nmNPs, presented in this study, demonstrates that nmNPs can easily penetrate the animal body, both of terrestrial and aquatic organisms and can be toxic to the CNS in vivo and to CNS-derived cells in vitro. Though a large and diverse group of nmNPs has been engineered and studied, they seem to share common mechanisms of toxicity. Adverse effects of nmNPs are usually associated with the generation of oxidative stress that leads to the malfunction of mitochondria, the activation of different signaling pathways, and subsequent activation of autophagy or apoptosis. Some nmNPs can, however, directly interact with cell membrane proteins, which leads to the activation of ion channel and flux of ion, e.g., calcium, from cellular milieu. The potential effects of nmNPs in the CNS are summarized in Figure 3.

## 8. Limitation of the Study and Future Directions

A general impression of non-metallic nanomaterials’ effects on the CNS after reading this review might be biased by the fact that the review is focused on the adverse effects of nmNPs. Thus, it must be understood that there is also a large number of publications describing little or no effects of nmNPs on the CNS. These publications have been omitted as being beyond the scope of this review. However, while limited toxicity might be a potential benefit of non-metallic nanomaterials, predisposing them to biomedical applications, it should be kept in mind that it can be harmful and there is a need to estimate the risk of its use, especially in medicine and diagnostics. While this review clearly shows a negative impact of nmNPs on the CNS, including mammals and non-mammalian vertebrates, their impact on human health in terms of long-term daily exposure is still unclear. Available data in regard to human exposure are very limited, and it is not possible to draw reliable conclusions. Thus, similar to the recently published report by the European Commission Directorate General for Environment on nanoplastic health impact [184], reporting standards should be developed for various nmNPs, which can penetrate different biological barriers and slip through filters more than other particles. This requires enormous work by nanotechnology scientists to conduct numerous experiments associated with the estimation of toxicity-tested materials. The report should include current quantification and assessment methods, the occurrence of NPs in the environment, their ecotoxicity, and what is most importantly their impacts on human health.

## Figures and Tables

**Table 1:** The effect of nmNPs (toxic or non-toxic) on CNS-derived normal cells in vitro.

Toxic Effect					
Surface Coating and/or NPs	Size, Concentration and Exposure Time		Model	Results	Ref.
**SILICA NPs**					
None	30 nm, 0.01, 0.1 and 1 mg/mL, 10 min, 30 min and 1 h		primary cultures of cortical neurons isolated from rats	Decreased neuron dendrite and induction of ROS production. Early stage apoptosis process and necrosis.	[80]
None	50, 100, 300 nm, 25–200 μg/mL, 24 h		microglia (N9 and BV-2), endothelial cells (bEnd.3), neuronal cells (HT22 cells)	Size- and cell type dependent cytotoxicity. Increased ROS production in microglia, decreased GSH level, lysosomal damage. Induction of release of IL-1β and N-terminal GSDMD, a marker of pyroptosis.	[81]
ICG/PCL- (PCL-SiNPs) or ICG/PLLA-coated SiNPs (PLLA-SiNPs)	90 nm (PCL-SiNPs) 95 nm (PLLA-SiNPs), 2.49 × 10^−7^ μg/mL to 24.9 × 10^−7^μg/mL, 2 and 24 h		brain endothelial cells and rat brain capillary endothelial cells (rBCEC4)	Time- and concentration-dependent decrease in cell viability, but not proliferation, differentiation. No inflammation process.	[82]
None	150–200 nm, 4 × 10^10^ NPs/mL to 7 × 10^10^ NPs/mL, 24 h		primary rat microglia	Increased production of ROS and RNS. Decrease in TNFα gene expression and cyclooxygenase-2 gene. Increased IL-1β cytokine release.	[23]
None	47; 49; 55; 500; 2000 nm, 20 µg/mL, 30 min and 4 h		neuronal cell lines (GT1–7 and GN11 cells)	Long lasting but reversible calcium signaling, independ on NP internalization.	[83]
None	509.3, 356.0, 469.0 nm, 100 and 200 µg/mL, 24 and 48 h 7 days		HEK293 cells and primary mouse cortical neurons	α-Synucleinopathy in HEK293 cells and neurons.	[84]
**CARBON NPs**					
Carboxylated and aminated MWNTs	200–300 nm, 5–100 μg/mL, 24 h		primary neuronal and glial cells isolated from fetal rat frontal cortex and striatum	Decreased microglia viability at concentration of 20 μg/mL or higher. Release of NO.	[19]
MWNTs	85–115 nm, 190 and 295 ppm, 24 and 48 h		NeuroScreen-1 (NS-1) cells	Reduced viability, apoptosis. Mitochondria depolarization and disruption of membranes.	[85]
MWNTs	5 to 15 nm, 0.6 μg/mL, 70 h		microglial cells (BV-2)	Dose-dependent cell division arrest and apoptosis. Perturbation of cell migration and phagocytosis.	[86]
Single-wall carbon nanohorns	80 to 100 nm, 16 h		microglial cells (N9 and BV-2)	Proliferation inhibition, promotion of apoptosis. Perturbation of cell cycle.	[87]
**DENDRIMERS**					
G4 and G4-C12 PAMAM	1 to 100 nM, 24 h		primary neuronal cultures	Apoptosis and cell death.	[25]
PAMAM-NH_2_ G4 (PAMAM-NH_2_) or (PAMAM-NH_2)_ modified with sodium carboxylate (PAMAM-S.C.)	26.1, 38.2, 104.8 nm (PAMAM-NH_2_) 45.3, 173.4, 267.7 nm (PAMAM-SC), 0.3, 1, 3, and 10 µg/mL, 72 h		human neural progenitor cells cultured as a 3D neurosphere model	Inhibition of neurosphere growth, cell proliferation and neuronal migration. Down-regulation of early growth response gene 1, insulin-like growth factor-binding protein 3 and tissue factor pathway inhibitor (TFPI2).	[88]
PAMAM G4, bare or modified with 4-carbomethoxypyrrolidone surface groups	10, 40, 80, 120, 200 µM, 24 h		embryonic mouse hippocampal cells (mHippoE-18)	Minor toxicity. No effect on ROS production nor mitochondrial membrane potential.	[89]
Cationic carbosilane dendrimer G2 loaded with FITC	100 nM, 24 and 48 h		human primary astrocytes	Cell death.	[90]
None	0.1 mg/mL, 30 min		neurons and glial cells from rats	Increase in intratracellular Ca^2+^ flux and mitochonria depolarization and impaired oxidative metabolism in pyramidal neurons and astrocytes.	[39]
**SOLID LIPID NPs**					
PLGA NPs with a peptide-binding transferrin receptor, loaded with siRNA	115 and 150 nm, 0.1; 1; 10; 100; 1000 µg/mL, 24 h		immortalized human cerebral microvascular endothelial cell line (hCMEC/D3 cell line)	Decreased metabolic activity of cells at the highest concentration.	[91]
**POLY(ETHYLENEGLYCOL)–POLY(LACTIDE) NPs**					
PLGA NPs coated with BSA	80 nm, 0.025 to 8.0 mg/mL, 4 h		capillary endothelial cell (BCEC)	Concentration dependent cytotoxicity.	[64]
**DNA NPs**					
DNA NPs modified with PEG	<60 nm, 1, 5, 10 μg/mL, 24, 48 and 72 h		rabbit and rat primary astrocytes	Toxicity.	[92]
**NANOGEL**					
PEG or PEG-PEI nanogel	<100 nm, 0.001; 0.01; 0.1; 1; 10 mg/mL, 2 h		bovine brain microvessel endothelial cells (BBMEC)	Neglectable cytotoxicity.	[43]
**NANOPLASTIC**					
Carboxylated polystyrene NPs	55 nm, 7.8–250 mg/L, 24 h		NE-4C neuroectodermal stem cells; primary brain cell cultures from mouse; microglia; brain vascular endothelial cell cultures	LDH leakage of neuronal cells. Microglial cells were able to internalize carboxylated polystyrene nanoparticles by phagocytosis.	[93]
Polystyrene NPs	100 nm, 100–200 μg/mL		GES-1, different primary brain cells	Reduced cell viability and defective neuronal development, reactive astrocytosis in astrocytes	[94]
**Non-toxic Effect**					
**Surface Coating and/or NPs**		**Size, Concentration and Exposure Time**	**Model**	**Results**	**Ref.**
**SILICA NPs**					
ICG/PCL-rhodaminedoped SiNPs (PCL-NPs) and ICG/PLLA-rhodamine-doped SiNPs (PLLA-NPs)	90 nm (PCL-NPs) 95 nm (PLLA-NPs), 2.6 × 10^9^, 5.2 × 10^9^ and 2.6 × 10^10^ (PCL-NPs/mL) [2.9 × 10^9^, 5.8 × 10^9^ and 2.9 × 10^10^ (PLLA-NPs/mL), 6 and 24 h		rat primary hippocampal culture	No change in the release of IL-1β and TNFα.	[24]
PEG and lactoferrin	26; 53 and 105 nm, 1 to 10 nM, 24 h		in vitro blood−brain barrier (BBB) model consisting of three distinct types of cells: endocytes, pericytes, and astrocytes	Lactoferrin enhanced efficiency of NPs transport across the BBB. The cell viability at a level over 93%.	[95]
Mesoporous silica NPs bare or coated with a PEG-PEI block copolymer	50 to 240 nm, 10, 20, 50 μg/mL, 36 h		rat brain endothelial cells (RBE4)	No toxicity up to 50 μg/mL. No damage to BBB.	[96]
**CARBON NPs**					
Carboxylated and aminated MWNTs	20–30 nm, 5–100 μg/mL, 24 h		primary neuronal and glial cell populations isolated from fetal rat frontal cortex and striatum	Neurons from both brain regions were generally not affected by exposure to NPs. The viability of mixed glia was not reduced in frontal cortex.	[19]
Prestine and pluronic F127 coated MWNTs	20–30 nm, 3.5, 17.5 μg/mL, 24 h		neurons and glia	No toxicity and no apoptosis after co-treatment with Pluronic F127 and MWNTs. No toxicity after treatment with prestine MWNTs.	[97]
Amino-functionalized MWNTs	18.9, 20 and 50 µg/mL, 24 and 72 h		primary porcine brain endothelial cells (PBEC) and primary rat astrocytes	No statistically significant toxicity.	[32]
MWNTs modified with plasmid DNA	20 nm, 2.5 μg of MWNTs, 15, 24 and 48 h		microglial cells (BV-2)	No detrimental effect on proliferation or cytokine production.	[21]
Acid-oxidized MWNTs	5, 7 and 8 µm, 1–10 µg/mL, 24 h		microglial cells (N9)	Confirmed NP internalization without any effect on viability, pro-inflammatory cytokine release or NO production.	[22]
Graphene modified with poly-L-lysine	7 days		mouse hippocampal culture model	No effect on cell viability and morphology. No effect on neuron growth.	[98]
Nanodiamonds	114.7 nm, 1, 5, 10, 25, 50, 100, 250 µg/mL, 2–3 days		mouse hippocampal neurons and mouse dorsal root ganglion neurons	Low neuronal toxicity but disturbances in neuronal morphogenesis.	[37]
**DENDRIMERS**					
CMCht/PAMAM	22.0 to 30.7 nm, 200 µg/mL, 1, 2, 6, 12, 15, 18, 24 and 48 h		hippocampal neuron cultures and cortical glial cells	No cytotoxicity.	[28]
CMCht/PAMAM dendrimer NPs	45 and 250 nm, 200–400 µg/mL, 1, 6, 12, 24, and 48 h, 7 days		human immortalized astrocytes (hTERT/E6/E7)	Low level of cytotoxicity (20% of decrease in metabolic activity) after long-term exposures (7 days).	[99]
**POLY(LACTIDE-CO-GLYCOLIC ACID) NPs**					
PLGA loaded with curcumin, and modified with g7 peptide	200–250 nm, 10, 20, 40 μM, 24 h		primary hippocampal neurons from rats	Lack of cytotoxicity. No significant increase in apoptotic nor necrosis.	[34]
PLGA loaded with curcumin	200 nm, 0.001, 0.01, 0.1, 0.2, 0.5, 5, and 50 μM, 24 h		neurospheres in culture and neural stem cells	Eenhanced proliferation of the neural stem cells at doses as low as 0.001 μM, with the highest proliferation at 0.5 μM. 0.5 μM of NPs were non-cytotoxic.	[100]
PLGA curcumin loaded, modified with PEG and conjugated with B6 peptide	less than 150 nm, 50, 100, 200, and 500 µg/mL, 24 h		HT22 cells	No effect on cell viability.	[35]
PLGA modified with PEG and phage-displayed peptides	121.46 nm, 0.1; 0.5, 1; 2.5 mg/mL, 4 h		endothelial cells (bEnd.3)	No cytotoxicity.	[101]
**POLYMER NPs**					
TEB NPs, bare	25 nm, 50, 100, 250, 500, 800 ng/mL, 24 h		endothelial cells (bEnd.3)	No obvious effect on cell viability.	[102]
**SOLID LIPID NPs**					
PLGA functionalized with a transferrin receptor or peptide mimicking transferrin receptor and loaded with siRNA	115, 150 nm, 0.1; 1; 10; 100; 1000 µg/mL, 24 h		immortalized human cerebral microvascular endothelial cell line (hCMEC/D3 cell line) brain capillary endothelial cell line (BCEC)	No toxicity.	[91]
**SERUM ALBUMIN NPs**					
R-flurbiprofen	284.4 nm, 25, 50, 100 and 200 μM, 48 h		chinese hamster ovary (CHO) cells stably transfected with mouse Ab precursor protein 695	No effect on cell viability.	[103]
Polysorbate 80 or with attached Apolipoprotein E	249 nm, 0.1, 1 and 2 mg/mL, 24 h		endothelial cells (b.End3)	Stimulation of cell viability.	[67]
**OTHERS**					
Gelatin-siloxane NPs modified with SynB peptides-cell penetrating peptides	194.55 nm, 100–600 µg/mL, 4 and 12 h		primary cultures of rat brain capillary endothelial cells	Good biocompatibility with brain capillary endothelial cells.	[104]
Biodegradable polymersomes conjugated with cationic albumin	95 nm, 0.25, 0.5, 1, 2, 4, 8 mg/mL, 60 min		endothelial cells (bEnd.3)	Only little toxicity (viability above 85%).	[75]

ICG—indocyanine green (dye), PLGA, PLG—poly(lactic-co-glicolic acid), PCL—poly(caprolatone), PLLA—poly(l-lactic acid), PEG/PLA—poly(ethylene glycol)/poly(lactide) copolymer, PAMAM—polyamidoamine, PEG-PEI—poly(ethylene glycol)/poly(ethylenimine), TEB—poly[Triphenylamine-4-vinyl-(P-methoxy-benzene)], CMCht—carboxymethylchitosan, MWNTs—multi-walled carbon nanotubes, SWCNTs—single-walled carbon nanotubes.

**Table 2:** The effect of nmNPs (toxic or non-toxic) in CNS-derived cancer cell in vitro.

Toxic Effect								
Surface Coating and/or NPs		Size, Concentration and Exposure Time		Model		Results		Ref.
**SILICA NPs**								
(ICG)/(PCL)-rhodamine-doped NPs and Si-ICG/PCL/ PLLA rhodamine-doped NPs		90 and 95 nm, 2.6 × 10^9^, 5.2 × 10^9^, 2.6 × 10^10^ PCL-NPs/mL and 2.9 × 10^9^, 5.8 × 10^9^, 2.9 × 10^10^ PLLA-NPs/mL, 6 and 24 h		neuroblastoma (SH-SY5Y)		Neurite outgrowth was not significantly altered. Reduction in neuronal differentiation.		[24]
Aminopropyltriethoxysilane		92.6 nm, 25–200 μg/mL, 24 h		neuronal cells (PC12)		The accumulation of cells in the G2/M phase at a concentration of 100 and 200 μg/mL. Activation of the p53-mediated signaling pathway and apoptosis.		[65]
None		509.3, 356.0, 469.0 nm, 100 and 200 µg/mL, 24 and 48 h, 7 days		Neuroblastoma (SH-SY5Y)		Mitochondrial dysfunction, pathological autophagy and cell apoptosis. Oxidative stress.		[84]
**CARBON NPs**								
MWNTs modified with carboxyl groups		50 and 500 nm, 10 and 40 μg/mL, 24 h		neuroblastoma (N2a)		Up-regulation of nNOS level in cells via promoting nuclear translocation and acetylation of NF-κB. Increase in NO level.		[111]
MWNTs with pluronic F127 solution		5 μg/mL or 10 μg/mL, 3 days, 1 week, 2 weeks		Neuroblastoma (SH-SY5Y)		Higher concentrations of NPs (50 and 500 μg/mL) and longer incubation times (1 and 2 weeks) caused a decrease in the viability of cells.		[112]
SWNTs with DNA conjugated with different surfactants sodium dodecyl sulfate, sodium dodecylbenzene sulfonate and sodium cholate		0.5 mg/mL, 72 h		astrocytoma (1321N1)		NPs with SC did not affect cell morphology, proliferation, or growth. NPs with SDS and SDBS surfactants demonstrated irregular cell morphology, and they were toxic to cells.		[50]
Graphene layers and SWNTs		0.8–1.2; 0.01; 0.1; 1; 10; 100 μg/mL, 24 h		neuronal cells (PC12)		The generation of ROS after exposure of graphene layers. The upregulation of caspase 3 indicates an apoptosis of PC12 cells.		[113]
PEG coated CNTs		2.5–4.5 nm, 0.1; 1; 10; 100 μg/mL, 24 h		neuronal cells (PC12)		The decrease in metabolic activity and generation of ROS. NPs with PEG exhibited less cytotoxic potency than uncoated NPs.		[114]
Graphene, SWNTs and MWNTs coated with an ultrathin layer of gold/platinum		50 nm (graphene), ∼100 nm (SWCNTs), and 2–5; 10–15 nm (MWNTs), 0.5–500 ppm, 24 h		neuronal cells (PC12)		The significant dose-dependent decreases in the viability of cells. Graphene exerted adverse effects on the neural cells at a concentration over 62.5 ppm.		[115]
Fullerenes C60 and C70 functionalized with dextran polymer		30.29 nm		glioma (C6)		Dose-dependent reduction in cell viability. NPs affect the growth, proliferation, functional and phenotypic aspects of cells. The slight degeneration of actin filaments and cytoskeletal destruction. Lysosomal integrity as well as mitochondrial membrane potential was significantly affected. ROS level inside cells was slight.		[116]
**DENDRIMERS**								
(G7) PAMAM with amine, acetamide, and carboxylate end groups, with GM1-pyrene		4–8 nm, 100 nM, 200 nM, and 400 nM and 1 μM, 1 h		glioma (C6)		The cells exhibited a greater sensitivity to G5-NH_2_ and G7-NH_2_ exposure but no differential effect was observed as a function of the presence of GM1 in the membrane.		[53]
PAMAM		0–100 μg/mL 24 h		human glioma cell lines (87MG, U251MG, U118 and A172)		Toxic. Akt/mTOR pathway was involved in the initiation of dendrimers-induced autophagy. The autophagy process induced by dendrimers is mediated by intracellular ROS generation.		[109]
Complexed with siRNA		200 nm, 0.005 and 0.01 mM, 4 h		glioblastoma (U87)		Primary amine acetylation of dendrimers reduced their cytotoxicity.		[117]
Second generation amphiphilic polylysine dendrons with siRNA		3 μM 72 h		glioblastoma (U87 and C6)		Inhibition of the proliferation of two glioblastoma cell lines. Non-toxic for non-tumoural CNS cells. Mitochondrial depolarization and the increase in ROS production.		[118]
CMCht/PAMAM dendrimer		45 and 250 nm, 200–400 µg/mL, 1, 6, 12, 24, and 48 h, 7 days		GBM cel line (U87MG)		Only long-term exposures (7 days) induced low levels of cytotoxicity (20% decrease in metabolic activity).		[99]
**LIPID NPs**								
D-α-tocopheryl PEG 1000 succinate -coated solid lipid NPs with resveratrol		128.6 to 429.1 nm, 0–200 µg/mL, 72 h		glioma (C6)		High toxicity in cancer cells. The metabolic activity significantly decreased depending on the concentration of NPs.		[119]
Solid lipid NPs with retinoic acid and functionalized electrostatically with trimethyl chitosan		214 nm, to 500 µg/mL, 24 h		glioblastoma (U87MG)		Anti-tumor inhibitory effect by decrease in cell viability and the presence of cells in early and late apoptotic and necrotic phases.		[120]
D-α-tocopheryl PEG 1000 succinate (TPGS) coated liposomes with resveratrol		61–262 nm, 0–200 µg/mL, 72 h		glioma (C6)		High toxicity in cancer cells. The metabolic activity significantly decreased depending on the concentration of NPs.		[54]
Liposomes loaded with resveratrol PEGylated, modified with transferrin		230 nm, 12.5 μM to 200 μM, 24 and 48 h		glioblastoma (U87MG)		Toxic. Higher levels of apoptosis accompanied by activation of caspases 3/7. NPs with transferrin were more effective in inducing toxicity.		[121]
Liposomes and micelles with diatom microalgae-derived nanoporous biosilica		90.5 and 115 nm, 1.70 mg /mL, 1 h		neuroblastoma (SH-SY5Y)		Toxic. The viability was 9–10%.		[122]
PLGA lipid NPs conjugated with folic acid and ICG with resveratrol		104.5–121.1 nm, 5, 10, 50 and 100 μg/mL, 24 h		glioblastoma (U87)		No changes in viability rate. Apoptosis process.		[68]
**POLY(LACTIDE-CO-GLYCOLIDE) NPs, POLIMERIC NPs**								
PLGA, PLG -tocopheryl PEG NPs with resveratrol		135–222 nm, 0–200 μg/mL, 72 h		glioma (C6)		High toxicity in cancer cells. The metabolic activity decreased depending on the concentration of NPs.		[59]
TMC surface- PLGA, PLG NPs		150 nm, 0.025–8.0 mg/mL, 24 h		neuroblastoma (SH-SY5Y)		Cell viability was slightly reduced at higher concentrations.		[123]
Ephrin type-A receptor 3 (EPHA3) tyrosine kinase antibody-modified PLGA NPs		145.9 nm, 4 ng/mL, 48 h		glioma (C6)		NPs inhibited of cell growth.		[57]
Aptamer was conjugated to the surface of PEG-PLGA NPs		156 nm, 0.019, 0.038, 0.38, 0.76, 3.8, 12 and 24 mg/mL, 24, 48 and 96 h		glioma (C6)		The IC50 value was detected at a concentration of 1.5 µg/mL (24 h incubation time). The cytotoxicity is dependent on incubation time.		[124]
Polymeric NPs with curcumin		5 or 10 μM, 24 h		medulloblastoma, glioblastoma (DAOY, D283Med)		A dose-dependent decrease in cell growth via programmed cell death and cell cycle arrest. Dose-dependent reduction in expression of both the IGF ligands and IGF-1R. Reduction in total STAT3α protein levels and increase phosphorylation of STAT3 at Tyr 705.		[125]
Tf conjugated NPs, poly(lactide)-D-a-Tocopheryl PEG PLA-TPGS diblock copolymer		137.6 nm, 0.05, 0.50 and 2.50 µg/mL, 24, 48 and 72 h		glioma (C6)		Toxic. 50% of death cells was observed at a concentration of 5 µg/mL.		[58]
mPEG–PCL NPs with resveratrol		87.5 nm, 0–31 μM 48 h		glioma (C6)		A dose-dependent cytotoxicity against cells.		[55]
**DNA NPs**								
Highly PEGylated		43, 47, 59 nm, 1, 5, 10 µg/mL, 24 h		rat gliosarcoma cells (9L)		Toxic. The viability was significant decreased.		[92]
**CHITOSAN NPs**								
Lactoferrin		300, 800, 1200 µg/mL, 24 h		glioma 261		Results suggest that the cytotoxicity of lactoferrin and NPs on glioma is attributable to its cytoplasmic allocation.		[126]
**OTHERS**								
Cardamom extract-loaded gelatin NPs		<200 nm		glioblastoma (U87MG)		Apoptosis process and inhibition of the viability.		[127]
Lactoferrin-curcuminoid coated polysaccharide NPs based on chitosan hydrochloride/hyaluronic acid/PEG		210–240 nm, 0–10 µg/mL		Glioma (C6)		Loaded NPs showed 50% cytotoxicity at concentration of 8–10 µg/mL.		[56]
**Non-toxic Effect**								
**Surface Coating and/or NPs**	**Size, Concentration and Exposure Time**			**Model**	**Results**			**Ref.**
**SILICA NPs**								
None		10–20, 40–50 nm, and 90–110 nm, 0.24; 2.4; 24; 240; 2400 ppb, 24 h		glioblastoma (A-172)		NPs were not toxic but NPs alter the membrane permeability.		[105]
**CARBON NPs**								
PKH26-labeled MWNTs		200–400 μm length, 80 μg/mL 0–48 h		glioma (GL261) expressing eGFP		NPs did not affect to cells proliferation.		[21]
SWNTs with CpG oligodeoxynucleotides		5 µg/mL, 24 h		glioma (GL261) expressing eGFP and luciferase-		No toxicity in gliomas.		[110]
Protoporphyrin IX (PX)-modified oxidized mesoporous carbon nanospheres		90 nm, 0–50 µg/mL, 24 h		Neuroblastoma (SH-SY5Y)		No toxicity during a 24 h treatment. NPs with ultrasounds were protective through the decrease in Aβ-mediated cellular toxicity.		[128]
**DENDRIMERS**								
Sialic acid		0–15 µM 24 h		Neuroblastoma (SH-SY5Y)		Attenuation of Aβ induced neurotoxicity.		[129]
**POLY(LACTIDE-CO-GLYCOLIC ACID) NPs, POLIMERIC NPs**								
G-protein estrogen receptor (GPER-1) selective agonist was encapsulated in polymeric NPs and liposomes, coated with polyssorbate-80		84.36 nm, 1, 10, 100 mg/mL 15 h and 24 h for N2a; 12 h and 24 h for SHSY5Y		neuroblastoma (N2a and SHSY5Y)		NPs were not toxic to cells at 324 µg/mL, but were taken up by cells.		[51]
PLGA, PLG NPs with curcumin		101 nm, 0.5 µM, 1 h		human neuroblastoma (SK-N-SH)		Prevention of the phosphorylation of Akt and Tau proteins in cells after induction by H_2_O_2_. anti-inflammatory and antioxidant activities of NPs.		[49]
Rosmarinic acid- and curcumin-loaded polyacrylamide-cardiolipin- PLGA, PLG NPs with conjugated 83-14 monoclonal antibody		40 μg/mL, 24, 36 h		neuroblastoma (SK-N-MC)		The protective role of NPs in cells induced with Aβ peptide. NPs caused the recovery of pp38 and p-S202 expressions to normal levels.		[130]
Polymeric nanostructures sulfonated and sulfated NPs		40 nm, 1.6 µg/mL 24 h		neuroblastoma (SH-SY5Y)		The protective role of NPs in cells induced by toxic Aβ peptide through decreasing caspase-3 activity and increasing cell viability.		[131]
**LIPID NPs**								
Linear polyethyleneimine (LPEI)-*g*-PEG copolymer-based micellar nanoparticle with siRNA		<100 nm, 24 h		neuroblastoma (N2a)		No toxicity. The viability was not decreased.		[132]
Spongosome and cubosome lipid NPs co-encapsulate curcumin and fish oil, rich in ω-3 polyunsaturated fatty acids		100 and 400 nm, 300 and 500 nM, 24 h		neuroblastoma (SH-SY5Y)		The cytotoxicity of the blank and antioxidant-loaded nanocarriers was negligible. The protective effect in cells induced by H_2_O_2_.		[133]
**OTHERS**								
Chitosan NPs copolymerized with PLGA, PLG		110 nm, 40 µg/mL 24 h, 96 h		neuroblastoma (SH-SY5Y)		The protective effect in cells against toxicity induced by Aβ peptide.		[134]
Lactoferrin NPs with curcumin		43–60 nm, 2 µM 24 h		neuroblastoma (SK-N-SH)		The cells were rescued from rotenone-induced neurotoxicity after NPs treatment. Antioxidant activity of NPs.		[48]
Nanocurcumin with BSA		153 nm, 0–500 nM, 24 h		neuroblastoma (SH-SY5Y)		NPs prevented cell death induced by 6-hydroxydopamine. The reversion of decrement p-Akt/t-Akt ratio in cells.		[135]
Apolipoprotein E3 mediated poly(butyl) cyanoacrylate NPs containing curcumin		178 nm, 10, 100, 1000 nM, 24 h		neuroblastoma (SH-SY5Y)		The protective role in cells induced by Aβ peptide (antioxidant effect of NPs). The decrease in apoptotic cell population.		[136]
Natural brain penetration enhancer-modified		100–200 nm, 50 ng/mL, 4 h		glioma (C6)		NPs showed good biocompatibility and negligible cytotoxicity.		[60]

ICG—indocyanine green (dye), PLGA, PLG—poly(lactic-co-glicolic acid), PCL—poly(caprolatone), PLLA—poly(l-lactic acid), PEG/PLA—poly(ethylene glycol)/poly(lactide) copolymer, PAMAM—polyamidoamine, PEG-PEI—poly(ethylene glycol)/poly(ethylenimine), TEB—poly[triphenylamine-4-vinyl-(P-methoxy-benzene)], CMCht—carboxymethylchitosan, MWNTs—multi-walled carbon nanotubes, SWNTs—single-walled carbon nanotubes, TMC—trimethylated chitosan, Tf—transferrin, BSA—bovine serum albumin.

**Table 3:** The effect of nmNPs (toxic or non-toxic) in vivo. Mammalian species.

Toxic Effect							
Surface Coating and/or NPs		Size, Concentration and Exposure Time	Model			Results	Ref.
**SILICA NPs**							
SiO_2_NPs modified with aminopropyl-triethoxysilane		15 nm, 20 μg/rat, 1 day, 7 days	adult rats	Induction of the oxidative stress and an increased inflammatory response in the striatum. The decrease in neurotransmitter dopamine and the downregulation of tyrosine hydroxylase protein in the brain.			[65]
None		115 nm, 8 mg/kg, 1 and 2 months	male C57BL/6 N mice	Mood dysfunction and cognitive impairment and neurodegeneration-like pathology and synaptic changes via ERK activation.			[137]
None		6, 20 and 50 nm, 150 μg/mL, 28 days	Wistar male rats	SOD and CAT activity was increased in the brain. The MDA level was increased in the brain and degenerative changes in the nerve fibers.			[138]
None		509.3, 356.0, 469.0 nm, 5 μg/mL, 3 months	male transgenic mice expressing A53T human a-Syn (a-Syn A53T Tg mice)	NPs promoted PD-like pathology including α-Synuclein aggregation and dopaminergic neuronal degeneration. Mitochondria impairment, oxidative stress, autophagy dysfunction, and neuronal apoptosis.			[84]
None		50 and 500 nm, 20 mg/kg, 1 h, 2 h, 24 h and 28 days	mice	Degeneration of neurons. The regulation of apoptosis by regulating Bax and Bcl-XL expression. Elevation of the autophagic responses. NPs administered via the intranasal instillation route resulted in more severe brain lesions compared to the intravenous injection route.			[139]
None		40 and 80 mg/kg, 14 days	rats	NPs passed from the BBB into the brain. The reduction in the activity of SOD and CAT. Cellular morphological modifications, mitochondrial dysfunction, and oxidative stress.			[140]
**CARBON NPs**							
MWNTs modified with carboxyl groups		5–40 µg/mL, 2, 4, 7 days	C57BL/6J mice	The neurotransmitter level was decreased. The increase in the NOS release. High levels of nNOS expression in regions associated with regulation of sympathetic nerve activity.			[111]
MWNTs		5 mg/m^3^, 5 h	male Sprague–Dawley rats	Inhalation of NPs significantly changes the balance between sympathetic and parasympathetic nervous system.			[88]
MWNTs shortened by oxidation and functionalized with amino groups (oxMWNT-NH_3_^+^) or functionalized with amino groups (MWNT-NH_3_^+^)		20–30 nm, 500 ng/mouse, 30 days post-injection	female C57/Bl6 mice	The increase in pro-inflammatory cytokine gene expression. oxMWNT-NH_3_^+^ induced a higher expression of pro-inflammatory cytokines compared to MWNT-NH_3_^+^. In addition, oxMWNT-NH_3_^+^ induced higher expression of GFAP and CD11b.			[70]
MWNTs		49 nm, 10 or 40 μg/mouse, 4 h	male C57BL/6J mice	Neuroinflammatory responses depend on the disruption of BBB integrity.			[141]
SWNTs modified with PEG		10 to 1000; or 1000 to 10,000 nm, 0.5, 1.0, and 2.1 mg/mL, 24 h and 30 min	Wistar male rats	Impairment of fear memory retrieval. Lipid peroxidation in the hippocampus.			[142]
**DENDRIMERS**							
G4 and G4-C12 modified PAMAM		1 μM, 24 h	C57/BL6-j mice	Higher concentrations of G4-C12 PAMAM dendrimer were toxic and caused the apoptosis process, while G4 PAMAM accumulation did not show any sign of apoptosis. Low level of glial activation.			[25]
**OTHERS**							
Chitosan NP modified with Polysorbate 80		251 nm, 3, 10, 30 mg/kg, 0.5, 2, 4, 8, and 24 h, 7 days	Sprague-Dawley male rats	NPs can enter the brain and induce the apoptosis and necrosis of neurons, slight inflammatory response in the frontal cortex. The decrease in GFAP expression in the cerebellum.			[143]
Polibuthylcyanoacrylate NPs modified with Polysorbate 80 and polystyrene NPs modified with dalargin		200 nm, 13.5 mg/kg, 5 min	mice	The locomotor activity decreased.			[144]
Polystyrene NPs, COOH-modified		80, 100, 200 nm, 7 days	mice after the aerosol inhalation	NPs with a size of 80 nm can deposit in the brain of mice via aerosol inhalation triggering neuron toxicity and altering the animal behavior. Inhibition of aChE activities.			[145]
**Non-toxic Effect**							
**Surface Coating and/or NPs**	**Size, Concentration and Exposure Time**		**Model**		**Results**		**Ref.**
**CARBON NPs**							
MWNTs, NH_2_-functionalized		18.9 nm, [^111^In]- MWNTs (50 µg, 0.5 MBq) in 100 mL PBS,5 min, 30 min, 1 h, 4 h and 24 h	C57/Bl6 mice	NPs were present in both brain capillaries and parenchyma fractions. NPs are potential nanocarriers to use for the delivery of drugs.			[32]
MWNTs, coated with Pluronic F127		10–30 nm, 0.5 mg/mL, 3 days	mice	NPs provoked no damage to the overall organization of the brain.			[97]
MWNTs		~20 nm, 5 µg, 72 h	GL261-bearing mice	NP uptake occurred by tumor-associated macrophages. No toxicity to mice with glioma.			[146]
MWNTs, COOH-modified		40 nm, 1 mg/mL, 7, 28 days	BALB/C mice	The lack of pathological changes in the brain.			[147]
Fullerene C60		200–500 mg/kg, 1, 3, 6, 16, 30 and 160 h	rats	NPs were able to penetrate the BBB, but the toxicity was found to be quite low.			[148]
Fullerene C60		3.4 mg/kg, 10–12 month	mice	No health deterioration in mice. No significant effect on body weight, spontaneous locomotor activity, and grip strength. Fasting blood glucose and glucose tolerance are not affected. No changes in the blood parameters in mice. No significant influence of NPs on organ weight except for a higher kidney weight in males compared with females.			[149]
Nanodiamond		50 nm, 20 mg/kg, 28 days	mice	Very low NPs concentration was detected in the brain.			[150]
Nanodiamond		114.7 nm, 10 μL of NPs at a concentration of 100 mg/mL, 10 min	rats	NPs did not induce cytotoxicity in primary neurons from either central (CNS) or peripheral nervous system (PNS) and did not affect animal behavior.			[37]
Carbon nano-onion		15 nm, 10 mL per g body weight of 1.0 mg/mL of NPs, 4, 12, 24 h	FVB/N mice	Carbon nano-onion crosses not only through the BBB into the brain of leukoencephalopathy mice but also through the glioblastoma multiforme-induced mice.			[151]
**DENDRIMERS**							
PEG		1.9 nm, 55 mg/kg, 1, 4 and 24 h	cerebral palsy rabbits model, murine orthotopic model of glioblastoma	NPs were taken up by the brain and accumulated in the corpus callosum (white matter), hippocampus, and cortex. They fully penetrate and distribute throughout the solid tumor.			[107]
Modified with PEG, lactoferrin and DNA		50 µg/mouse, 2 h	Balb/c mice	Lactoferrin improved the NP uptake. NPs can be a potential non-viral gene vector to the brain via noninvasive administration.			[152]
Dendrimers and dendriplexes loaded with siRNA		15 mg/kg in 200μL PBS, 1 and 24 h	BALB/c mice	NPs were present inside the brain, but there was no specific brain histology alterations.			[90]
N-acetyl cysteine and valproic acid		∼5 mg/kg dogs, 48 h	dogs	NPs were not toxic but were present in the brain. Dendrimers with drugs improved neurological outcome in injured brain.			[153]
None		~6.7 and ~4.3 nm, 6 mg/kg, 48 h	dogs (brain injury model)	Generation 6 dendrimers showed extended blood circulation times and increased accumulation in the injured brain compared to generation 4 dendrimers, which were undetectable in the brain by 48 h after final administration.			[30]
Ethylenediamine-core		4 nm, 2.5 µg of dendrimer in 5 µL of PBS, 24 h	white rabbits	The high uptake of NPs into astrocytes and microglia cells.			[26]
**LIPID NPs**							
Liposomes modified with BSA		118.2–185.8 nm, 11 and 25 mg lipid /kg, 1, 3, 6 and 24 h	Wistar rats	NPs were able to move into brain tissue. NP uptake is dependent on the presence of BSA. NPs are a promising tool in drug delivery to the CNS.			[74]
Liposomes modified with PEG		40, 80, 200 nm, 1 mM, 1, 6, 24 and 48 h	rats	Liposomes accumulated in a subpopulation of perivascular cells within the brain dependent on charge and PEG coated. NPs are a promising tool in drug delivery to the CNS.			[154]
D-α-tocopheryl polyethylene glycol 1000 succinate (TPGS) coated liposomes with resveratrol		107.8, 212.5 and 262.3 nm, 2 mg/kg of resveratrol, 0, 0.083, 0.25, 1, 2, 4, 8, 12, 24, 36 and 48 h	Charles Foster rats	NPs showed high accumulation in the brain but are biocompatible and safe.			[54]
Liposomes with phospholipid, a polymer surfactant and cholesterol		<200 nm, 10 and 150 µL form 20 nM solution, 4 and 24 h	rats and C57/BL6 mice	NPs were delivered to myelinated peripheral nerves. The liposomes were presented in choroid epithelium, but not in myelinated white matter regions or in brain parenchyma. NPs are a promising tool in drug delivery to the CNS.			[155]
Micellar linear polyethyleneimine (LPEI)-*g*-polyethylene glycol (PEG) copolymer NPs		below 100 nm, 0.6%, 0.8% and 1.2% PEG grafting density in LPEI-*g*-PEG/Bace NPs, from 2 to 7 days	C57BL/6J mice	NPs did not impact on the activation of astrocytes and microglial cells in ipsilateral hippocampus of mice. No inflammation and no cytotoxicity in the brain. Normal morphology of astrocytes and microglia.			[132]
**POLY(LACTIC-CO-GLYCOLIC ACID) AND POLYMERIC NPs**							
PLGA, PLG NPs modified with a 7-aminoacid glycopeptide and with albumin		234 nm, 2 mg NPs, 2 h	C57BL/6 mouse	NPs were able to deliver low molecular weight molecules-albumin across the BBB in two murine models of lysosomal storage disorders. NPs are a promising tool in drug delivery to the CNS.			[156]
PEG/PLA NPs, modified with cationic BSA		80 nm, 60 mg/kg, 30 min	mice	NPs were observed in the lateral ventricle, third ventricle and periventricular region of the brain. NPs are a promising tool in drug delivery to the CNS with low toxicity.			[64]
PLGA, PLG NPs modified with glycopeptide (g7)		200 nm, 1 mL of NPs (8 mg) suspension, 3 h	Balb/c mice	NPs can enter to the brain. NPs are a promising tool in drug delivery to the CNS.			[157]
PLGA, PLG -tocopheryl PEG 1000 succinate blend NPs with resveratrol		175.5, 199.7 and 222.5 nm, 2 mg/kg of resveratrol, 0.25, 0.5, 1, 2, 4, 8, 12, 24, 36 and 48 h	Charles Foster rats	NPs showed high accumulation in the brain. NPs are a promising tool in cancer therapy, and they are safe.			[59]
PLGA, PLG tocopheryl PEG succinate diblock copolymer modified with transferrin		161.5, 121.6, 137.6 nm, 5 mL/kg, 4 h	Sprague-Dawley rats	NPs were present in the brain. NPs could be able to deliver imaging/therapeutic agents across the BBB.			[58]
12-amino-acid-peptide conjugated onto the surface of PLGA, PLG PEG NPs		121 nm, 30 µg/kg, 0.083 h, 0.25 h, 0.5 h,1 h, 2 h, 4 h, 8 h,12 h and 24 h	nude mice	Enhanced brain accumulation efficiency together with lower accumulation in the liver and spleen was observed in the mice intravenously. Injection with peptide- conjugated NPs compared with plain NPs, shows powerful brain selectivity. NPs could be able to deliver imaging/therapeutic agents across the BBB.			[101]
PLGA, PLG NPs modified with ephrin type-A receptor 3 tyrosine kinase antibody		145 nm, 0.5 mg/kg, 1, 2, 4, and 8 h	Sprague-Dawley rats	Anti-EPHA3-modified NPs showed high fluorescence intensity in the brain and effectively accumulated in glioma tissues. A significantly higher tumor cell apoptosis.			[57]
PLGA, PLG 1,2-distearoyl-glycerol-3-phospho-ethanolamine-N-[methoxy(PEG)-2000] ammonium salt hybrid NPs modified with curcumin		169 nm, 25 µM, 5 days	Wistar rats	Tumor size decreased significantly after 5 days.			[158]
PLGA, PLG NPs modified with polysorbate 80, poloxamer 188 and chitosan		250 to 400 nm, 100 mg/mL, 0, 15, 60, 120, 240 min	rats	NPs concentrations in the brain were increased by NP surface modification. NPs could be able to deliver therapeutic agents across the BBB.			[77]
PLGA, PLG NPs modified with cationic BSA		97, 98, 104 nm, 30 mg/ kg, 0.033 h and 1 h	BALB/c mice	The increase in surface density of the NPs enhanced the BBB permeability. NPs are a promising tool in drug delivery to the CNS.			[159]
PEG-poly(ε-caprolactone) polymersomes modified with cationic BSA		95 nm, 10 mg/kg, 0.5, 1, 2, and 4 h	Sprague-Dawley rats	NPs can enter to the brain and might be a promising carrier for drug delivery to the brain.			[75]
**ALBUMIN NPs**							
None		143 and 151 nm, 1 µL from 10 µg/mL rats and 100 mg/ kg mice, 1,7, 14 days	adult rats and mice	NPs located in different brain tissues but did not induce an inflammatory response. No locomotor, explorative, or cognitive function impairment.			[160]
Polysorbate 80 or with attached apolipoprotein E		249 nm, 200 μg of NPs/g body weight, 15 and 30 min	female SV 129 mice	The uptake was dependent on the presence of apolipoprotein E. NPs with apolipoprotein E were taken up less by animal brain cells. NPs are a promising tool in drug delivery to the CNS.			[67]
borneol, muscone and menthol		100–200 nm, 40 mg/kg, 1, 2, 4, 8, 12, and 24 h	BALB/c mice	Accumulate in glioma cells with a much higher targeting efficiency than that of transferrin-modified NPs.			[60]
**CHITOSAN NPs**							
Thymoquinone-encapsulated		between 150 and 200 nm, 500 μg of TQ/25 μL, 0.25, 0.5, 2, 4, 6, 24 h	Wistar rats	Taken up by brain tissue. NPs are a promising tool in drug delivery to the CNS.			[161]
**OTHERS**							
Poly(n-butyl cyanoacrylate) dextran polymers coated with polysorbate 80		48 nm, 200 µL from 60 mg/mL of NPs, 2 days	APP/PS1 mice (AD model)	No induction of non-specific BBB disruption. NPs collaborate with plasma apolipoprotein E to facilitate BBB crossing.			[66]
Cyanoacrylate NPs, PEGylated and coated with polysorbate 80 or poloxamine 908		137 nm, 30 mg/kg, 60 mg/kg, 30 min; 1 and 4 h	OF1 mice and rats dark agouti DA/R	NPs can penetrate into the brain. Localization in the ependymal cells of the choroid plexuses, in the epithelial cells of pia mater and ventricles and to a lower extent in the capillary endothelial cells of BBB. NPs are a promising tool in drug delivery to the CNS.			[78]
Polybutylcyanoacrylate NPs coated with polysorbate 80, loaded with curcumin		152 nm, 5 mg/kg, 0.033; 0.083; 0.25; 0.5; 1.25; 0.75; 1; 2; 3; 4; 8; h	mice	NPs inside the brain tissue. NPs are a promising tool in drug delivery to the CNS.			[162]
Polysaccharide NPs based on hyaluronic acid and chitosan hydrochloride, functionalized with lactoferrin and loaded with curcumin		>200 nm, 1.25 mg/kg, 24 h	ICR mice	Accumulated in the brain. The presence of lactoferrin improved the NP uptake. NPs can be potentially used for cancer therapy.			[56]
PLA NPs coated with T-80		162 nm, 8 mg/kg, 45 min	mice	Present in the brain. The specific role of T-80 coating on NPs in brain targeting. NPs are a promising tool in drug delivery to the CNS.			[69]
Polystyrene latex nanospheres		20, 100 and 1000 nm, 90–120 days	Fischer F344 rats	Low numbers of particles in the brain.			[71]
Polystyrene NPs COOH-modified and methoxy (MeO)-PEG amine (NH_2_)		60 nm, 2.5 mg/kg, 1 and 24 h	Fischer F344 rats	Diffusion in normal brain tissue. Delayed tumor growth following local administration, this effect was dependent on the presence of PEG.			[79]
PEG and polyethylenimine (complexes of oligonucleotides with 3H-labeled nanogel)		<100 nm, 200 µL, 1 h	mice	Accumulation in the brain. Nanogel is a promising system for the delivery of oligonucleotides to the brain.			[43]
Gelatin-siloxane NPs modified with SynB peptide		194 nm, 1 mL, 60 mg/kg, 0.5, 1, 2, and 4 h	nude mice and Sprague-Dawley rats	The presence of peptide improved the NP uptake. No toxicity.			[104]

PLGA, PLG—poly(lactic-co-glicolic acid), PCL—poly(caprolatone), PAMAM—polyamidoamine, MWNTs—multi-walled carbon nanotubes, SWNTs—single-walled carbon nanotubes.

**Table 4:** The effect of nmNPs (toxic) in vivo. Non-mammalian species.

Toxic Effect						
Surface Coating and/or NPs	Size, Concentration and Exposure Time		Model	Results		Ref.
**SILICA NPs**						
None	62 nm, 25, 50, 100, 200 µg/mL, 4–96 h	zebrafish embryos (*D. rerio*)			Embryonic developmental toxicity, resulted in persistent effects on larval behavior.	[173]
**CARBON NPs**						
Fullerenes aqueous suspended colloids (nC60)	0.5 ppm and 1 ppm, 48 h	juvenile largemouth bass			Lipid peroxidation in brain after 48 h of exposure.	[171]
Fullerenes (C60), long or short	MWNTs, SWNTs and 0.001 mg/L, 21 days	*D. rerio*			High level of alterations in the DNA/ RNA region, especially in the brain.	[172]
**POLYSTYRENE NPs**						
None	50 nm, 0, 1, 10, 100 and 1000 μg/L, 72 h	*Caenorhabditis elegans* used as a model organism to evaluate the neurodevelopmental toxicity			Significant inhibition in body length, survival rate, head thrashes, and body bending. The increase in ROS production. The lipofuscin accumulation, apoptosis and decrease in dopamine contents. pink-1 gene was involved in the polystyrene NPs-induced neurotoxicity. NPs at concentration of 100 μg/L caused up-regulation of 89 genes and down-regulation of 56 genes regulated differently expressed genes correlated with biological function of cuticle development and molting cycle. The neurodevelopmental toxicity and oxidative stress responses induced by NPs were regulated by dpy-5 and rol-6.	[175]
NH_2_-modified, positively charged	25–330 nm, 0.005 g/L to 0.150 g/L, 24 h	*Daphnia magna*			The alterations in behavioral patterns, decreased brain mass and morphological changes in the cerebral gyri.	[167]
None	<100 nm, 100 mg/L NPs, 15 days	*Cyprinus carpio*			Degrees of necrosis, fibrosis, changes in blood capillaries, tissue detachment, edema, degenerated connective tissues, and necrosis in large cerebellar neurons and ganglion cells. The activity of acetylcholinesterase (aChE) significantly decreased after exposure to 100 mg/L.	[168]
None	50 and 200 nm, 10–10,000 parts per billion, 5 days	larval zebrafish (*D. rerio*)			Accumulation in the tissues of larval zebrafish, alteration of their transcriptome, and changes in behavior and physiology. Potentially decreasing organismal fitness in contaminated ecosystems.	[174]
None	50 nm, 1 mg/L, till 120 h post fertilization	*D. rerio*			Neurotoxicity. Suppression of locomotion activity.	[176]
Fluorescent NPs	51 nm, 0.1, 1, 10 ppm, until 120 h post fertilization	*D. rerio*			Accumulation in the brain and alteration of larval behavior.	[177]
Fluorescent NPs	70 nm, 0.5, 1.5, 5 ppm, 7 days, 30 days and 7 week	*D. rerio*			Alteration of neuro-behavior and neurotransmitter regulation.	[178]
Fluorescent NPs	20 nm, 270 ppm, 120 h	*D. rerio*			NPs can reach brain, oxidative damage and apoptosis.	[179]
None	100 nm, 1, 10, 100, 1000 µg/L, Prolonged exposure:from L1-larvae to adult day-1	*C. elegans*			Induction of neurodegeneration of D-type GABAergic motor neurons. Alteration of forward and backward movement.	[180]
None	0.1, 0.5, 1, 2 and 5 μm, 1 mg/L, 3 days	*C. elegans*			Excitatory toxicity on locomotive behavior. Damage to cholinergic and GABAergic neurons.	[181]
None	0,11 µm, 0.005, 0.05, 0.5, 5 and 50 mg/L, 96 h	Mediterranean mussel (*Mytilus* *galloprovincialis*)			Changes in gene expression. *Cholinesterase* inhibition in hemolymph.	[169]
NH_2_-modified, positively charged	50 nm, 0.1, 1.0, 3.0 and 10.0 mg/L, 48 h up to 14 days	Brine shrimp (*Artemia fransiscana*)			Cholinesterase inhibition. Glutathione S-Transferase and catalase decreased.	[170]
None	40 nm, 10 mg/L, 7 days	Japanese rice fish (*Oryzias latipes*)			Particle presence in the brain, suggesting penetration of BBB.	[166]
None	0,1 µm, 1, 10 and 100 μg/L, 1–14 days	Red tilapia (*Oreochromis* *niloticus*)			Particle presence in brain tissue. Inhibition of aChE activity in the brain.	[182]
NH_2_-modified, positively charged	100 mg/L, 64 days	Crucian carp (*Carassius carassius*)			Particle were present in the brain and caused brain weight loss. Behavioural changes and enlarged cerebral gyri.	[167]
None	50 nm, 1 mg/L, 3 days	Zebrafish, larvae (*D. rerio*)			Particle were present in head, gills and muscle. Decrease aChE activity.	[183]

MWNTs—multi-walled carbon nanotubes, SWNTs—single-walled carbon nanotubes.

## Data Availability

Not applicable.
